# Optimizing a reliable ex vivo human blood model to analyze expression of *Staphylococcus epidermidis* genes

**DOI:** 10.7717/peerj.9295

**Published:** 2020-06-15

**Authors:** Susana Brás, Ângela França, Nuno Cerca

**Affiliations:** Centre of Biological Engineering (CEB), Laboratory of Research in Biofilms Rosário Oliveira (LIBRO), University of Minho, Braga, Portugal

**Keywords:** Bacterial survival, *Staphylococcus epidermidis*, Human blood ex vivo model, Volume of human blood, Gene expression

## Abstract

Human blood is often used as an ex vivo model to mimic the environment encountered by pathogens inside the host. A significant variety of experimental conditions has been reported. However, optimization strategies are often not described. This study aimed to evaluate key parameters that are expected to influence *Staphylococcus epidermidis* gene expression when using human blood ex vivo models. Our data confirmed that blood antimicrobial activity was dependent on initial bacterial concentration. Furthermore, blood degradation over time resulted in lower antimicrobial activity, with a 2% loss of leukocytes viability correlating with a 5-fold loss of antimicrobial activity against *S. epidermidis*. We further demonstrated that the volume of human blood could be reduced to as little as 0.18 mL without affecting the stability of gene expression of the tested genes. Overall, the data described herein highlight experimental parameters that should be considered when using a human blood ex vivo model for *S. epidermidis* gene expression analysis.

## Introduction

*Staphylococcus epidermidis* is a commensal inhabitant of healthy human skin and mucosae that can cause important infections, such as medical device-associated bloodstream infections (Otto, 2009). Due to the clinical relevance of these infections, it is important to understand the strategies employed by *S. epidermidis* to evade the host immune system response. In order to better comprehend how *S. epidermidis* adapts to the host, gene expression studies are often conducted under conditions that try to mimic the in vivo environment (Loza-Correa et al., 2019). Human blood ex vivo models have contributed to a better understanding of how pathogens survive in human blood, by evaluating the transcriptional response during incubation in human blood, as well as, by exploring host-pathogen interactions. These models are relatively easy to implement, have been reported in many clinically relevant microorganisms, including *S. epidermidis* (França et al., 2014; Qin et al., 2017), *Staphylococcus aureus* (Malachowa et al., 2011), *Neisseria meningitidis* (Echenique-Rivera et al., 2011; Nolte et al., 2002) *Streptococcus agalactiae* (Mereghetti et al., 2008), *Enterococcus faecalis* (Vebo et al., 2009) and *Candida albicans* (Fradin et al., 2005; Fradin et al., 2003; Hunniger et al., 2014).

However, the implementation of these models lacks often do not describe optimization steps and, not surprisingly, a significant variety of experimental set-up conditions have been reported (Fradin et al., 2003; França et al., 2014; Hedman et al., 2012; Qin et al., 2017). Because different experimental designs can greatly influence experimental outcomes, we became interested in evaluating key parameters that can compromise *S. epidermidis* gene expression studies when using a human blood ex vivo model. To achieve this goal, we evaluated the initial bacterial concentration and the volume of human blood in the co-incubation assays, as well as leukocyte viability after blood collection, and assessed bacterial survivability and gene expression under a human ex vivo blood model.

## Material and Methods

### Bacterial strains and growth conditions

*S. epidermidis* PT12003, isolated from a patient with a central catheter after stomach surgery (Freitas et al., 2018), was used in this study. One single colony was inoculated into two mL of Tryptic Soy Broth (TSB) (Liofilchem, Teramo, Italy) and incubated overnight at 37 °C and at 120 rpm (ES-20 Shaker-Incubator, Biosan, Riga, Latvia). Planktonic cultures were started by adjusting the optical density at 640 nm (OD_640 nm_), to 0.050 (±0.005) in 10 mL of TSB and grown, in a 25 mL flask, for 6 h at 37 °C and at 120 rpm. The suspension was then washed once and resuspended in 0.9% NaCl. Before further experiments, the OD_640 nm_ of bacterial suspension was adjusted to 2.7 in order to obtain 1 ×10^9^ CFU/mL.

### Human blood collection

Peripheral blood was collected by venipuncture from Portuguese healthy adult volunteers with an age range between 25–40 (7 female and 4 male donors), not taking antibiotics or anti-inflammatory medication within the previous 14 days. Blood was drawn using one of the following anticoagulant tubes: K_3_EDTA (Vacuette, Greiner Bio-one, Kremsmünster, Austria), sodium citrate (Vacuette, Greiner Bio-one, Kremsmünster, Austria) or lithium heparin (Becton Dickinson, NJ, USA). Blood was collected under a protocol approved by the Institutional Review Board of the University of Minho (SECVS 002/2014 (ADENDA)), which is in strict accordance with the Declaration of Helsinki and Oviedo Convention. All donors gave written informed consent to have blood taken.

### The influence of anticoagulants on bacterial growth

In order to explore the effect of different anticoagulants on bacterial growth, TSB (Liofilchem, Teramo, Italy) was added to the different blood collection tubes and was gently inverted 5 to 8 times, before being transferred to a 25 mL flask. Planktonic bacteria obtained as described above was inoculated into TSB with the different anticoagulants, at a final concentration of 10^7^ CFU/mL. A negative control was included by inoculating bacteria in TSB without anticoagulants. Bacterial suspensions were incubated for 24 h at 37 °C and at 120 rpm (ES-20 Shaker-Incubator). Bacterial growth was determined by CFU quantification by taking aliquots every 2 h. The aliquots were serially diluted and plated on TSB agar using the (Miles, Misra & Irwin, 1938) and incubated at 37 °C. At least two independent experiments with two technical replicates were performed.

### The ability of bacteria to survive in human blood

For the evaluation of the ability of bacteria to survive in human blood, 0.1 mL of different concentrations of exponentially growing bacteria, were added to 0.9 mL of human blood in two mL tubes, to obtain final concentrations of 10^8^, 10^7^, 10^6^, 10^5^ or 10^4^ CFU/mL and incubated at 37 °C, at 80 rpm, for up to 8 h. Four and 8 h after incubation, the enumeration of bacteria was determined by CFU counting, as described above. The number of CFU/mL immediately (i.e., less than 2 min) after incubation with blood was used as a control to calculate the percentage of survival. This experiment was performed five independent times, using blood from five different donors.

### Viability of human blood leukocytes over time

Human blood leukocytes viability after collection was used as an indicator of blood degradation over time. Whole blood was collected and incubated for up to 8 h at 37 °C and 80 rpm (PSU-10i, Biosan). At time points 0 (immediately after collection), 4 h and 8 h, two mL of whole blood was collected and incubated with five mL of red blood cells (RBC) lysis buffer (Alfa Aesar, Karlsruhe, Germany). The suspension was mixed by carefully inverting the tubes and then incubated at room temperature for 10 min. The reaction was stopped by adding 15 mL of phosphate buffered saline (PBS) (Gibco, MA, USA). Leukocytes were harvested by 10 min centrifugation at 300 g and 4 °C, and a new RBC lysis cycle was performed to lyse residual red blood cells. Leukocytes were then suspended in 0.5 mL of PBS and cells viability determined through flow cytometry (EC800, Sony Biotechnologies Inc, CA, USA), using propidium iodide staining (5µg /mL, Sigma, MO, USA). This experiment was performed three independent times, using blood from different donors. Representative flow cytometry plots are presented in Fig. S1.

### The impact of time after blood collection on bacterial survival in human blood

Whole blood was collected and an aliquot (0.9 mL) was immediately taken and mixed in a two mL tube with 0.1 mL of *S. epidermidis,* to obtain a final bacterial concentration of 10^5^ CFU/mL, and incubated at 37 °C and 80 rpm for 4 h. The remaining blood was kept under the same temperature and agitation conditions for 4 h. After this time, a new 0.9 mL aliquot of blood was taken and a second incubation with 0.1 mL of *S. epidermidis* was performed for another 4 h, in a new two mL tube. Bacteria was quantified by CFU counting, as described above. This experiment was performed three independent times, using blood from three different donors.

### *S. epidermidis* gene expression assays

Three unrelated genes were selected as probes for assessing gene expression stability: *SERP_RS11970*, *SERP_RS10985* and *SERP_RS08870*. Two different experimental conditions were tested: (i) the utilization of different anticoagulants on the collection tubes and (ii) the reduction of the volume of blood used during the co-incubation assays (total incubation volume of one mL, 0.6 mL, 0.5 mL, and 0.2 mL). Blood samples were transferred into two mL tubes and, then, bacteria were added to each tube to obtain a final concentration of 10^8^ CFU/mL. The tubes were incubated for 2 h at 37 °C and 80 rpm (PSU-10i). After the co-incubation period, samples were sonicated for 5s at 33% amplitude (Cole-Parmer 750- Watt Ultrasonic Homogenizer 230 VAC, IL, USA) to lyse eukaryotic cells. Total RNA isolation, complementary DNA synthesis (cDNA) and quantitative PCR (qPCR) were performed as previously optimized (França et al., 2012), with minor modifications. In brief, after mechanical and chemical lysis of bacterial cells, total RNA was purified using EZNA total RNA kit (Omega Biotek, GA, USA). Genomic DNA was degraded by DNase I (Thermo Scientific, MA, USA) and cDNA synthesized, from 200 ng of total RNA, by RevertAid M-MuLV reverse transcriptase (Thermo Fisher Scientific) and using random primers (NZYTech, Lisboa, Portugal) as priming strategy. Finally, qPCR was prepared by mixing 2 µL of 1:100 diluted cDNA with 5 µL of Xpert Fast SYBR (Grisp, Porto, Portugal), 0.5 µL of each forward and reverse primers at 0.5 µM and 2 µL of nuclease-free water. The run was completed in a CFX96™ thermal cycler (Bio-Rad, CA, USA) with the following cycling parameters: 3 min at 95 °C followed by 40 cycles of 5 s at 95 °C and 25 s at 60 °C. The primers used were designed using Primer3 software (Koressaar & Remm, 2007; Untergasser et al., 2012) and synthesized at Metabion (Steinkirchen, Germany). Primers sequences, size of the amplicon and reaction efficiency are presented in Table 1. The quantification of the transcripts for each gene under study was determined using 16S rRNA as reference gene and by applying the delta C_q_ method (E^ΔCq^), a variation of the Livak method (Livak & Schmittgen, 2001), where ΔC_q_ = C_q_ (reference gene) −C_q_ (target gene) and E is the experimentally determined reaction efficiency. Reaction efficiencies were determined using the dilution method (Pfaffl, 2004) at 60 °C.

**Table 1:** List of primers used for the quantification of gene expression by qPCR.

**Gene**	**Primer sequence (5′-3′)**	**Product size** **(base pair)**	**Efficiency** **(%)**
***SERP_RS00125*** ***(16S rRNA)***	Fw: GGGCTACACACGTGCTACAA Rv: GTACAAGACCCGGGAACGTA	176	97
***SERP_RS11970***	Fw: CAGGCATTGAACTTCCCAAT Rv: AATTCGGGGGCATATTTAGG	109	103
***SERP_RS10985***	Fw: ATGATTTTAGTGCTATCCCTGACT Rv: CACTAATTGCAAGATCATTTTCTG	102	110
***SERP_RS08870*** ***(sepA)***	Fw: TCTTAAGGCATCTCCGCCTA Rv: GTCTGGTGCGAATGATGTTG	196	97

### Statistical analysis

Statistical analysis was carried out with GraphPad Prism Version 6 Trial (CA, USA). For comparisons among different groups one-way or two-away ANOVA, with Tukey’s comparisons test, were used when appropriate (the tests used are detailed in the figure caption). *P* < 0.05 was considered significant.

## Results and Discussion

Human blood ex vivo models have been developed to mimic bloodstream infections as an affordable alternative to in vivo models (Echenique-Rivera et al., 2011; Malachowa et al., 2011; Qin et al., 2017). During human blood collection, anticoagulants need to be used to prevent blood clotting. It has been pointed out that different anticoagulants may influence the experimental outcome (Freitas et al., 2008; Strobel & Johswich, 2018). The most commonly used anticoagulants are heparin, citrate and EDTA (Engstad, Gutteberg & Osterud, 1997). Citrate and EDTA prevent blood from clotting through binding free calcium ions (Strobel & Johswich, 2018), while heparin inhibits coagulation by enhancing the activity of antithrombin III (Engstad, Gutteberg & Osterud, 1997). Herein, pilot experiments were initially performed to determine the best anticoagulant for *S. epidermidis* gene expression analysis. Thus, the influence of heparin, citrate, and EDTA was evaluated on bacterial growth and transcription levels of the selected genes. As shown in Fig. S2, EDTA, but not the other tested anticoagulants, inhibited bacterial growth in the first 8 h, eventually leading to bacterial death, after 24 h of incubation. A similar effect was reported before in some strains of *N. meningitidis*, when using citrate as anticoagulant (Strobel & Johswich, 2018) but in our experimental setup, we did not observed any inhibitory effect regarding *S. epidermidis* growth. Interestingly, when analyzing bacterial gene expression no significant differences were found among the different anticoagulants tested. Taken into consideration (i) these pilot results, (ii) the availability and (iii) price difference between the anticoagulants tested, and previous experimental results (França et al., 2014; Franca et al., 2016) we selected heparin for the remaining the experiments.

**Figure 1:**
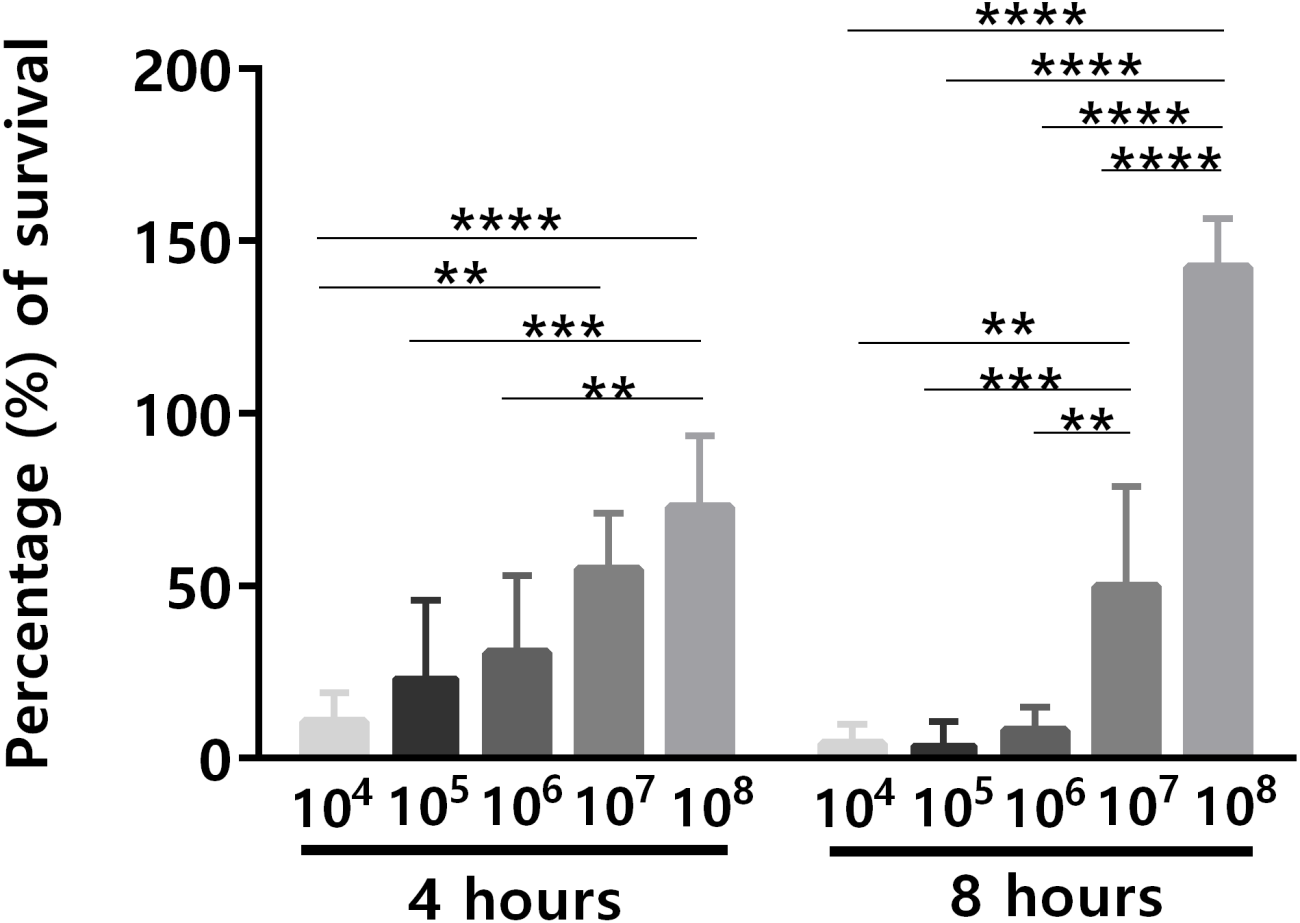
The effect of initial bacterial concentrations on the ability of *S. epidermidis* to survive in human blood after 4 h and 8 h of incubation. The bars represent the mean plus standard deviation of five independent experiments, performed with five different donors. Statistical analysis was performed using two-way ANOVA and Tukey’s multiple comparisons test. Significant differences between 10^8^ and the other bacterial concentrations are depicted with ^∗∗^*p* < 0.01; ^∗∗∗^*p* < 0.001; ^∗∗∗∗^*p* < 0.0001.

An important issue related to human blood ex vivo models is the bacterial concentration used. Although the quantity of microbes present in human blood during bacteremia is estimated to be up to 10^4^ CFU/mL (Opota et al., 2015), often higher concentrations of bacteria have been used in human blood ex vivo models (Askarian et al., 2017; França et al., 2014; Qin et al., 2017), mainly due to the lack of sensitivity of many experimental methods to assess lower bacterial concentrations (Bacconi et al., 2014; Machado et al., 2013). For instance, it is known that for the analysis of the transcriptomic response of bacteria, the initial bacterial concentration needs to be significantly higher, to ensure a sufficient amount of RNA for downstream applications (Hedman et al., 2012). To investigate the effect of different initial bacterial concentration on the ability of *S. epidermidis* to survive in human blood, different bacterial concentrations (10^4^ to 10^8^ CFU/mL) were used for the bacterial survival assays. After 4 h and 8 h of co-incubation, the percentage of bacterial survival was determined (Fig. 1). Since it is known that there is a significant source of experimental variability when working with human samples, due to the inherent traits of the donors such as age (Eady et al., 2005), gender (Whitney et al., 2003) and the proportion of different blood cell populations (Cobb et al., 2005), this experiment was performed with blood from five different donors, to increase the significance of our results. Not surprisingly, the ability of *S. epidermidis* to survive in human blood was cell concentration dependent: the lower the inoculum, the higher the percentage of bacterial killing by human blood (Fig. 1). Interestingly, after 4 h of co-incubation, cell death was observed in all tested bacterial concentrations but after 8 h of co-incubation, the higher concentration inocula presented higher cell density than at time zero. This suggests that over time, and when the bacterial inoculum was 10^8^ CFU/mL, blood lost some antimicrobial activity leading to bacteria growth.

**Figure 2:**
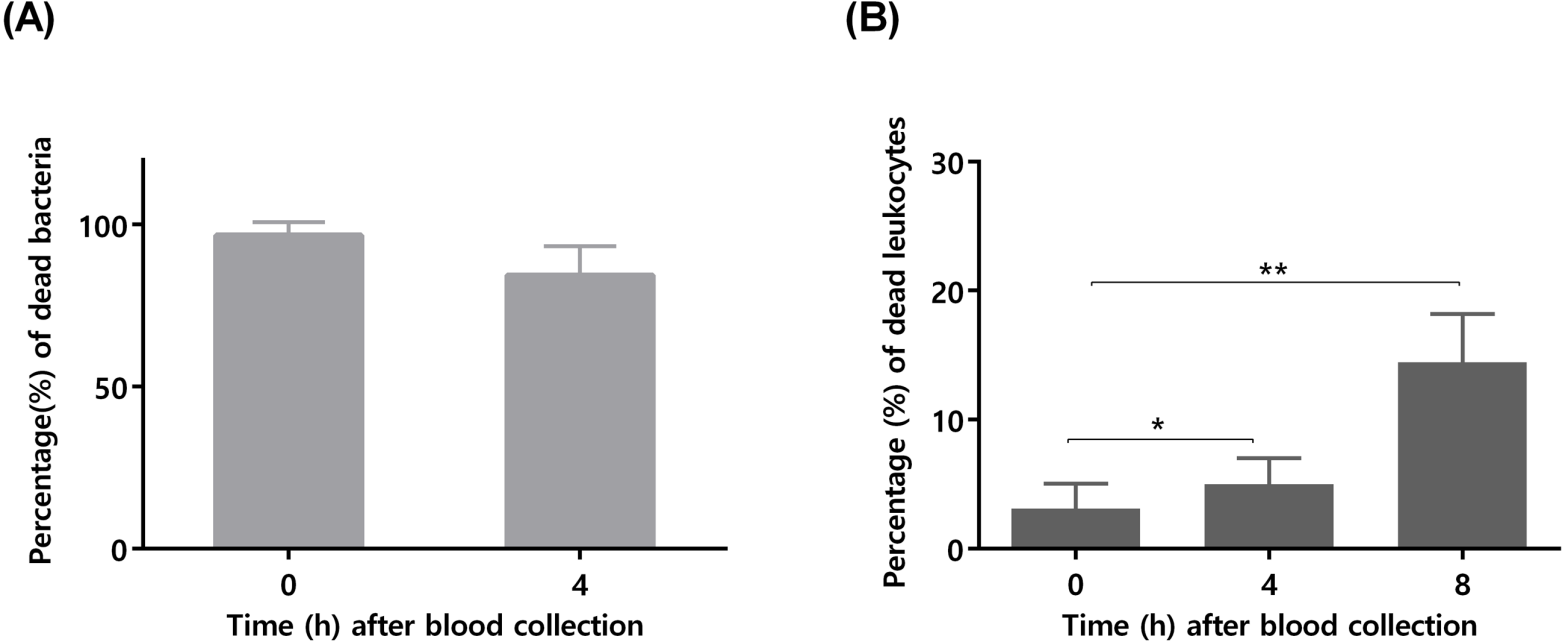
The influence of time after blood collection on bacterial survivability and leukocyte viability. (A) Bacterial survivability after 4 h of co-incubation with blood. Bacteria was added to blood immediately after collection or 4 h post-blood collection. Bacterial survivability was determined by CFU counting. The bars represent the mean plus standard deviation of three independent experiments, performed using three different donors. (B) Viability of human blood leukocytes after collection. Leukocytes viability was assessed by flow cytometry immediately, 4 and 8 h after blood collection, before utilization in the co-incubation assays. The bars represent the mean plus standard deviation of three independent experiments, performed using three different donors. Statistical analysis was performed using one-way ANOVA and Tukey’s multiple comparison test. ^∗^*p* < 0.05. ^∗∗^*p* < 0.01.

To confirm that longer blood incubation periods would result in lower antimicrobial activity, a second experiment was performed. For this assay, we selected a bacterial concentration of 10^5^ CFU/mL, taken in consideration the significant killing observed after 4 h of incubation (Fig. 1). As shown in Fig. 2A, the co-incubation of *S. epidermidis* with human blood after 4 h, resulted in different bacterial killing rates, depending if the blood was used immediately or 4 h after collection: while 97% of bacterial was killed if blood was used immediately, only 84% of death was observed if we used blood 4 h after collection. It is well described that blood antimicrobial activity against *S. epidermidis* involves important components such as complement and leukocytes (Le, Park & Otto, 2018). As such, to evaluate blood degradation, we assessed leukocytes viability right after blood collection, and also 4 and 8 h after collection. As shown in Fig. 2B, right after blood collection, 3% of leukocytes were already dead. This fact may be related to the process of collection and processing time of human blood (Ferrante & Thong, 1980). The results also showed that 4 h after blood collection, cell death rate was slightly increased to 5% and then to 15% after 8 h. Interestingly, a 2% reduction in leukocyte viability (from 3 to 5%) was correlated with 13% reduction in antimicrobial activity (from 97% to 84%). However, it should be noted that blood degradation over time will likely affect other important components that were not quantified herein, such as complement (Nordahl et al., 2004). As such, based on these results, it’s not possible to determine which exact mechanism involved in blood degradation contributed to the effective loss of antimicrobial activity over time. Nevertheless, our data clearly confirms that longer waiting times after blood collection will contribute to blood degradation. This fact is important to consider when planning ex vivo experiments with blood, as it may have substantial consequences on the results obtained, especially if a higher bacterial inoculum is needed, such as when performing RNA-sequencing (França et al., 2014; Franca et al., 2016; Malachowa et al., 2011; Qin et al., 2017). Some studies have used human blood ex vivo model with incubation periods up to 24 h (Nolte et al., 2002). However, as shown by our results, 24 h is an extensive period of incubation which may yield higher blood degradation and, as such, should be avoided when determining bacterial survival studies in human blood. Blood degradation can occur when research laboratories are not physically close to human blood collecting centers, and this should also be taken into consideration when planning experiments using human blood. A simple way to reduce this obstacle is to maintain the blood using gentle agitation (Afonso et al., 2010).

**Figure 3:**
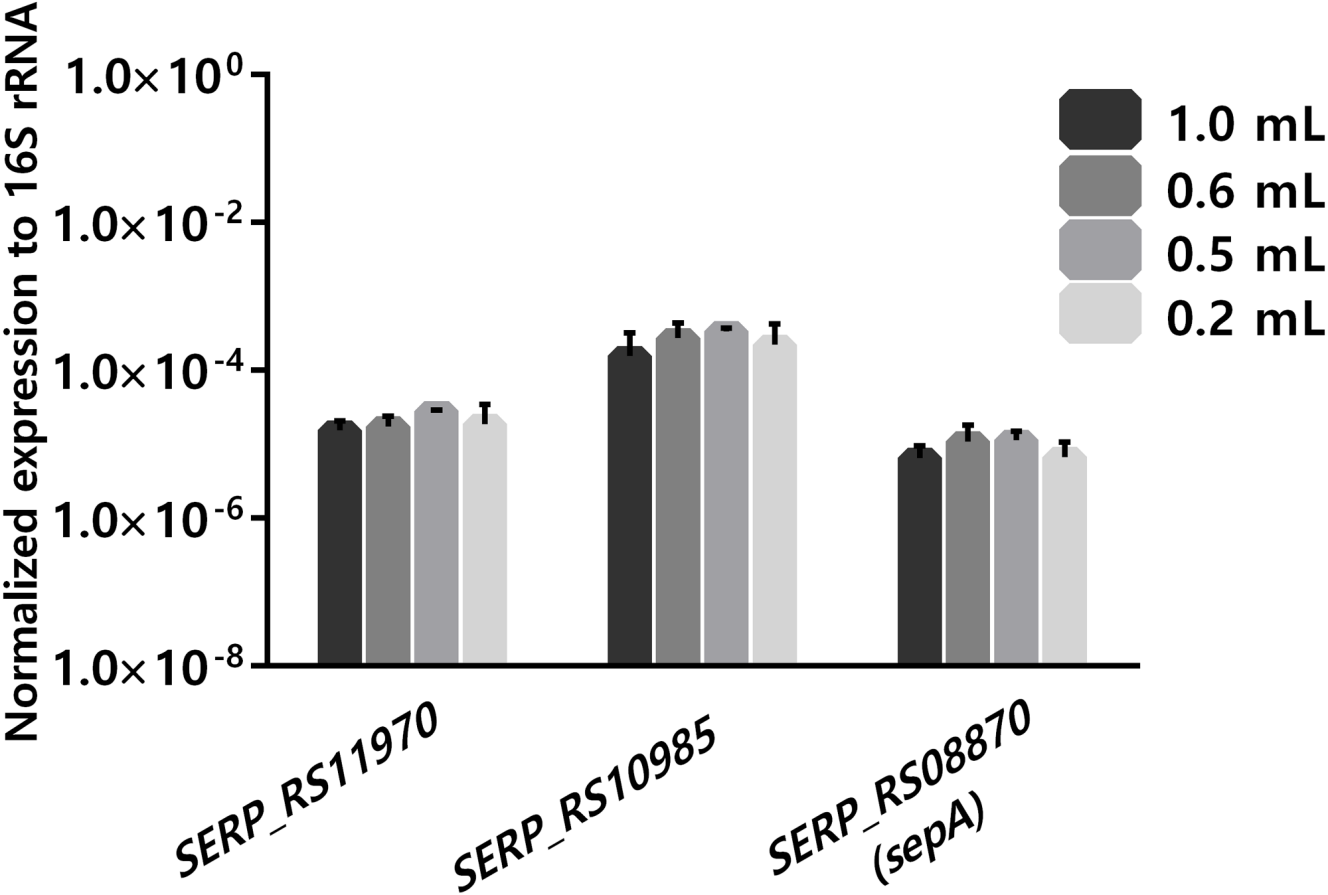
The influence of using different volumes of human blood in co-incubations assays on the stability of transcription levels of *SERP_RS11970*, *SERP_RS10985* and *SERP_RS08870* genes. Bacteria was added to each tube to obtain a final concentration of 10^8^ CFU/mL and then the tubes were incubated for 2 h at 37 °C. The bars represent the mean plus standard deviation of normalized expression of three independent experiments, performed with three different donors. Statistical analysis was performed, respectively, using one-way ANOVA and Tukey’s multiple comparisons test.

Another important practical aspect when considering using human blood as an ex-vivo model is the limitation of blood availability. As such, the ability to reduce the volume of blood per experiment, without compromising the results, is of interest. When analyzing several published gene expression studies, it was observed that different volumes of human blood, ranging from 0.2 mL to 80 mL per experiment, have been reported (Echenique-Rivera et al., 2011; Hedman et al., 2012; Hunniger et al., 2014; Mereghetti et al., 2008; Qin et al., 2017). As the long-term goal of our research group is to assess global transcriptomic changes occurring with *S. epidermidis* gene expression, using ex vivo blood models, there was interest in determining if reducing the volume of blood in the co-incubation assays down to 0.18 mL had a detrimental effect on the stability of gene transcription. The starting volume was 0.9 mL, in order to compare with our previous data obtained using RNA-seq (França et al., 2014). For this assay, we selected a bacterial concentration of 10^8^ CFU/mL to ensure a sufficient amount RNA that would be needed for future RNA-sequencing studies (Hedman et al., 2012). The transcription of three unrelated genes was assessed. One of the selected genes was *sepA* (*SERP_RS08870*), which codifies a protease that plays an important role in bacterial immune invasion through the degradation of antimicrobial peptides produced by the host (Lai et al., 2007). The two other selected genes were *SERP_RS11970*, a gene that codifies a major facilitator superfamily and *SERP_RS10985*, a universal stress protein. As shown in Fig. 3, no significant differences were found in the expression of the selected genes using, in any of the different volumes of human blood tested. Noteworthy, a volume of blood as low as 0.18 ml per reaction could be used without impacting the transcription of the selected genes. Nevertheless, we acknowledge that a limitation of this study was the fact that the selected genes had relative expressions (to the 16S ribosomal RNA) between ∼10E−3 and ∼10E−7. While this is a very large dynamic range, we can’t exclude the possibility that very low expressing genes (≤10E−8) could potentially be affected differently.

## Conclusion

There remains a great deal of work to be done in clarifying the factors that contribute to *S. epidermidis* survival and adaptation to blood, which contribute to the evasion from the host immune system. The improvement of human blood ex vivo models will contribute to standardization of experimental conditions and produce a more reliable experimental setup and, consequently, contribute to obtain results with higher clinical relevance. The findings from the study are of technical importance for future studies since it highlights key parameters that should be considered when using human blood as an ex vivo model for the analysis of the gene expression of *S. epidermidis,* in particular, the possibility of using low volume of blood per reaction, without compromising the experimental results, at least in regard to the parameters tested herein.

##  Supplemental Information

10.7717/peerj.9295/supp-1 Figure S1 Representative dot plots and histograms showing the gating strategy used to select the white blood cells population and evaluate its viability overtime White blood cells were separated from contaminating red blood cells and cells debris having in consideration their size and internal complexity characteristics. The viability of white blood cells was analyzed using propidium iodide (5 µg/mL) immediately **(A)**, 4 **(B)** and 8 **(C)** hours after blood collection.

10.7717/peerj.9295/supp-2 Figure S2 The effects of the anticoagulants on *S. epidermidis* growth (A) and on transcription levels of *SERP_RS11970*, *SERP_RS10985* and *SERP_RS08870* genes (B) Growth curves** are shown in CFU/mL obtained at different time points. The growth curves correspond to the mean ±standard deviation of two independent experiments. Statistical analysis was performed using two-way ANOVA and Tukey’s multiple comparisons test. ^∗∗∗^*p* < 0.001, ^∗∗∗∗^*p* < 0.0001 comparatively to the bacterial growth in TSB. The bars represent the mean plus standard deviation of two independent experiments. Statistical analysis was performed using two-way ANOVA and Turkey’s multiple comparisons test.

10.7717/peerj.9295/supp-3 Supplemental Information 1 Raw data for Fig. 1

10.7717/peerj.9295/supp-4 Supplemental Information 2 Raw data for Fig. 2

10.7717/peerj.9295/supp-5 Supplemental Information 3 Raw data for Fig. S2

10.7717/peerj.9295/supp-6 Supplemental Information 4 Raw data for Fig. S3

## Additional Information and Declarations

### Data Availability

The following information was supplied regarding data availability: Raw data is available in the Supplementary Files.
