# Supplementary figures and images for: Optimizing a reliable ex vivo human blood model to analyze expression of Staphylococcus epidermidis genes
Source: PeerJ. 2020 Jun 15;8:e9295. doi: 10.7717/peerj.9295 (PMC7301895; doi:10.7717/peerj.9295)

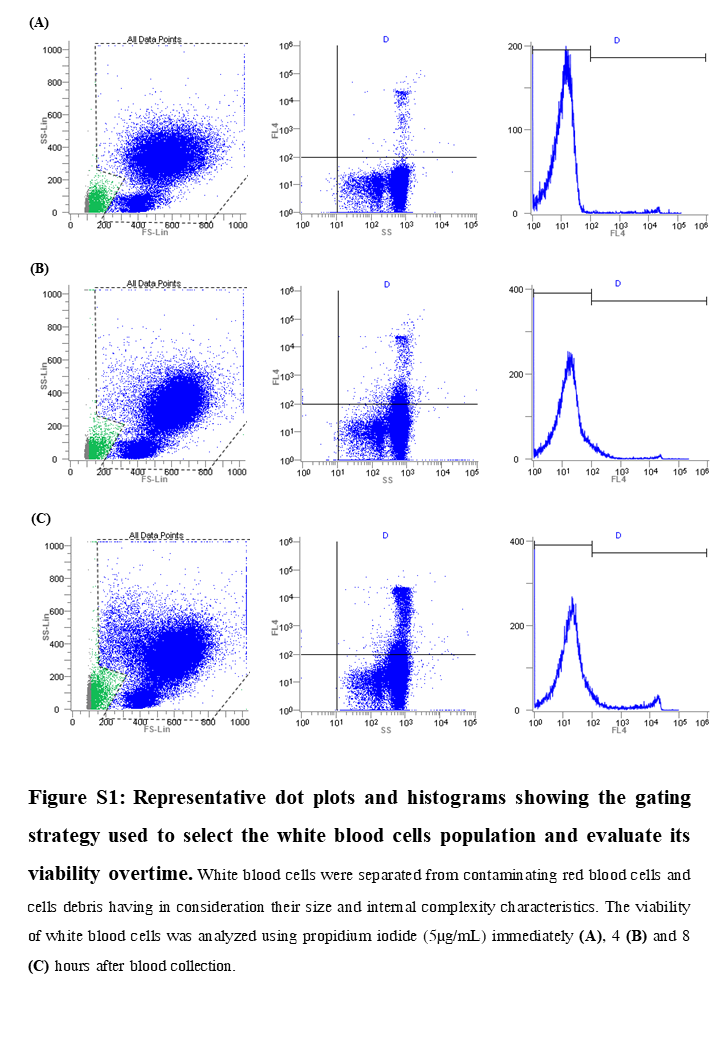

Supplement: Figure S1 — White blood cells were separated from contaminating red blood cells and cells debris having in consideration their size and internal complexity characteristics. The viability of white blood cells was analyzed using propidium iodide (5 µg/mL) immediately (A), 4 (B) and 8 (C) hours after blood collection. [file peerj-08-9295-s001.png]

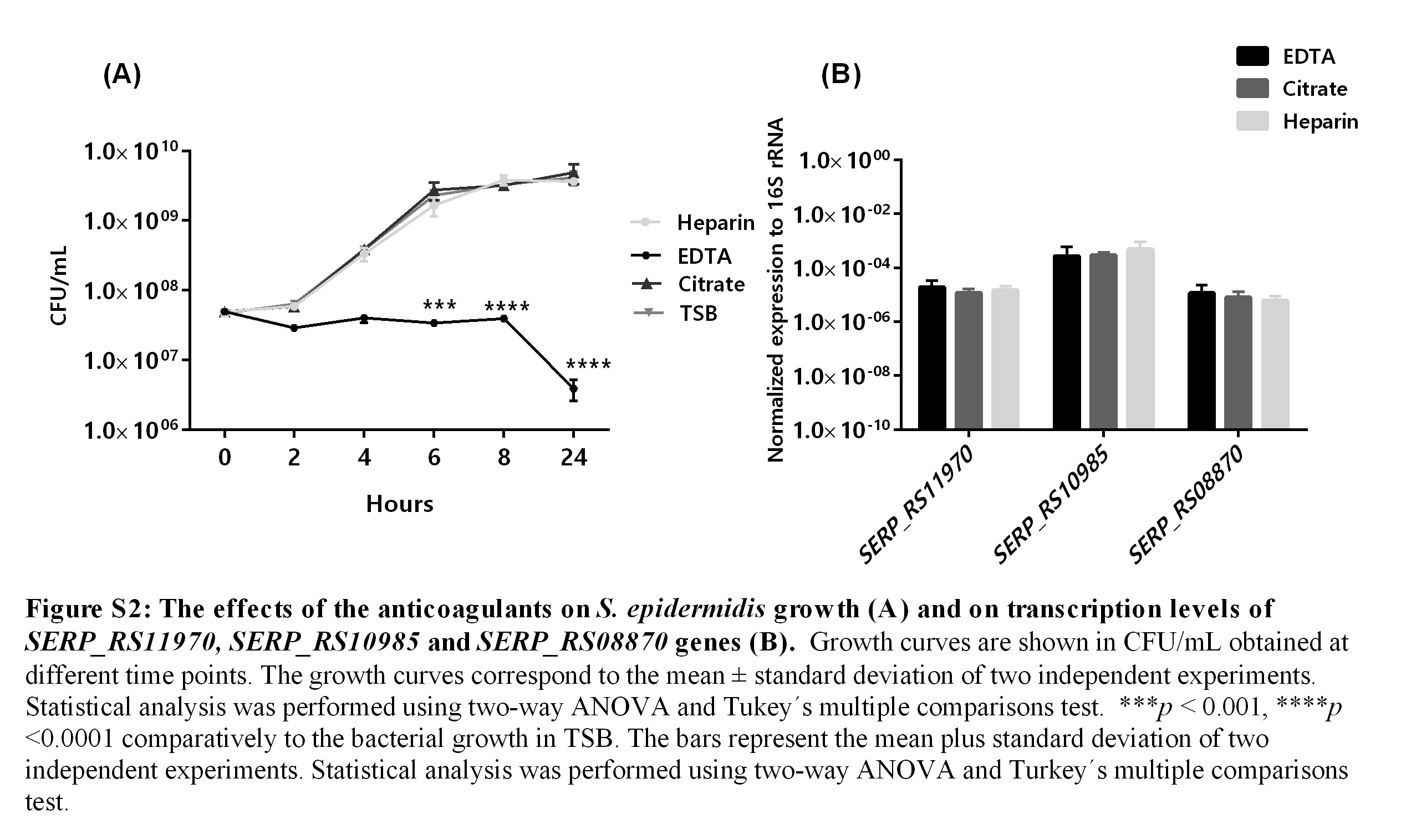

Supplement: Figure S2 — Growth curves are shown in CFU/mL obtained at different time points. The growth curves correspond to the mean ±standard deviation of two independent experiments. Statistical analysis was performed using two-way ANOVA and Tukey’s multiple comparisons test. ∗∗∗p < 0.001, ∗∗∗∗p < 0.0001 comparatively to the bacterial growth in TSB. The bars represent the mean plus standard deviation of two independent experiments. Statistical analysis was performed using two-way ANOVA and Turkey’s multiple comparisons test. [file peerj-08-9295-s002.png]
